# Mindful eating is associated with a healthier plant-based diet in the NutriNet-Santé study

**DOI:** 10.1038/s41598-025-02195-5

**Published:** 2025-06-06

**Authors:** Pauline Paolassini-Guesnier, Marion Van Beekum, Emmanuelle Kesse-Guyot, Julia Baudry, Rebecca Shankland, Angélique Rodhain, Alice Bellicha, Christophe Leys, Serge Hercberg, Mathilde Touvier, Benjamin Allès, Sandrine Péneau

**Affiliations:** 1 Center of Research in Epidemiology and StatisticS (CRESS), Nutritional Epidemiology Research Team (EREN), Université Sorbonne Paris Nord and Université Paris Cité, Inserm, INRAE, CNAM, Bobigny, 93017 France; 2 Desbrest Institute of Epidemiology and Public Health, IDESP UMR UA11 Inserm, Université Montpellier, Montpellier, France; 3 Montpellier Research in Management (MRM), University of Montpellier, Place Eugène Bataillon-CC 19001-bâtiment 15, Montpellier, 34095 France; 4 Laboratoire DIPHE (Développement, Individu, Processus, Handicap, Education), Université Lumière, Lyon 2, France; 5 University Institute of France, Paris, France; 6 Faculty of Psychology, Educational Sciences, and Speech and Language Therapy, Université Libre de Bruxelles, Avenue Franklin Roosevelt, 50-CP191, Brussels, 1050 Belgium; 7 Public Health Department, Avicenne Hospital, Assistance Publique-Hôpitaux de Paris, Bobigny, France

**Keywords:** Nutrition, Plan-based diets, Vegetarian, Vegan, Mindful eating, Epidemiology, Nutrition, Public health, Behavioural ecology

## Abstract

Scientific evidence suggests that mindful eating (ME) may be effective for promoting healthy plant-based diets. However, data are scarce. This cross-sectional study investigated the association between ME and plant-based diets. In 2023, 13,768 participants from the NutriNet-Santé cohort completed the Mind-Eat Scale, at least three 24-hour dietary records, and a food choices questionnaire. The contribution of plant-based foods was evaluated using the Plant-based Diet Index (PDI), the healthy PDI (hPDI), and the unhealthy PDI (uPDI). Multivariable linear and logistic regressions were performed to analyze the association between ME, the PDI, hPDI, and uPDI scores, the proportions of meat, fish, and dairy products in the diet, and diet groups, adjusting for socio-demographic and lifestyle factors. ME was positively associated with PDI (β = 1.19; 95%CI: 0.98, 1.41) and hPDI (1.00; 0.76, 1.24), and negatively with uPDI (-0.48; -0.70, -0.27), meat (-0.63; -0.76, -0.50), and dairy (-0.86; -1.14, -0.58) consumption. Individuals with higher ME levels were more likely to be lower meat eaters (OR = 1.13; 95%CI: 1.04, 1.23), pesco-vegetarians (1.56; 1.33, 1.83), vegetarians (2.19; 1.57, 3.05), or vegans (1.35; 1.24, 1.48). Participants with higher ME levels were more likely to follow healthy plant-based diets, suggesting ME could help to promote a reduction in animal product consumption. Longitudinal studies are needed to continue exploring this relationship.

**Clinical Trial Registry**: The NutriNet-Santé study was conducted in accordance with the Declaration of Helsinki, and all procedures were approved by the Institutional Review Board of the French Institute for Health and Medical Research (IRB Inserm n◦ 0000388FWA00005831) and the Commission Nationale de l’Informatique et des Libertés (CNIL n◦ 908450 and n◦ 909216). Electronic informed consent was obtained from all participants. Written informed consent by electronic means for adults, and for legal representant for children < 18 years old. The study was registered at clinicaltrials.gov as #NCT03335644 (https://clinicaltrials.gov/study/NCT03335644?locStr=Bobigny,%20France&country=France&state=%C3%8Ele-de-France&city=Bobigny&term=Cohort%20Study&rank=6).

**Supplementary Information:**

The online version contains supplementary material available at 10.1038/s41598-025-02195-5.

## Introduction

The benefits of a plant-based diet on both health and the environment are now firmly established^1–3^. Specifically, higher consumption of plant-based foods has been associated with a lower risk of mortality among adults^3^, a lower risk of type 2 diabetes (T2D)^4^, and a lower risk of coronary heart disease^5^. Individuals following vegetarian or vegan diets have also been found to have a 12% lower mortality risk from cerebrovascular diseases and a 16% lower mortality risk from circulatory diseases compared with omnivores^6^. Individuals who follow a pesco-vegetarian diet have also been shown to have better health than meat eaters^6^. In addition to health benefits, diets high in plant-based foods have been shown to have a lower environmental impact through lower land use, a reduction of water use, and a reduction of emission of nitrogen and phosphorus^3,7^. Particularly, vegetarian and vegan diets have been associated with the greatest reductions in greenhouse gas emissions and land use^8^, with vegetarian diets also showing the greatest reduction in water use^9^. Given the positive health and environmental benefits of plant-based diets, it is crucial to identify strategies for promoting these diets and understanding the factors that influence consumer choices. Mindful eating (ME) could be a promising approach in this regard.

Mindful eating can be broadly defined as paying attention to the eating experience with all the senses (seeing, tasting, hearing, smelling, and feeling) and observing the emotional and physical reactions that occur before, during, and after the eating experience without judgment or reaction^10,11^. ME may positively affect food intake by increasing internal physical cues to eat and decreasing emotional and external cues to eat^10^. Some countries, such as Canada^12^ and Germany^13^, have incorporated the concept of ME into their dietary guidelines. However, research exploring the potential association between overall ME and plant-based food consumption remain limited. The available data focused on ME sub-scores and showed that participants with higher scores for the ME sub-dimensions “health of the planet” and “awareness and appreciation for food” consumed more plant-based foods, particularly healthy options^14^. In contrast, participants who scored higher on “non-judgmental awareness” were more likely to follow an unhealthy plant-based diet^14^. Most available literature has concentrated on the broader concept of mindfulness and has demonstrated a positive correlation between mindfulness and sustainability through pro-environmental conduct^15,16^ and sustainable consumption^17^. These promising results highlight the need for further research examining the relationship between overall ME and diverse indicators of a plant-based diet. Specifically, it is important to explore how ME relates to the consumption of both plant-based and animal-based foods, as well as pesco-vegetarian, vegetarian, and vegan diets, which have notable environmental impacts. Additionally, examining how ME influences the intake of healthy and unhealthy plant-based foods is crucial, as it could help mitigate environmental impacts while reducing the risk of chronic diseases. However, it is important to note that not all plant-based foods are healthy. Many are ultra-processed^18^, high in refined carbohydrates and trans fats, and low in vitamins and minerals^19^.

The aim of this large population-based cross-sectional study was therefore to investigate the associations between ME (total score and sub-dimensions) and the contribution of plant-based foods (overall, healthy and unhealthy) to the diet, proportions of meat, fish, and dairy consumption, and the participants’ diet groups (higher meat eaters, lower meat eaters, pesco-vegetarians, vegetarians, vegans).

## Methods

### Study population and design

Participants were volunteers aged 15 years or older from the NutriNet-Santé study, which is a large ongoing French web-based prospective cohort launched in May 2009^20^. The overall aim of this study is to investigate the relationship between nutrition and health, as well as the determinants of health and eating behaviors. At inclusion and every year thereafter, participants complete a set of online questionnaires to assess their diet, anthropometric measures, physical activity, socioeconomic and demographic data, lifestyle characteristics, and health status. In addition, they complete monthly questionnaires related to determinants of eating behaviors, nutritional status, and specific health-related aspects. This study was conducted according to guidelines laid down in the Declaration of Helsinki. All procedures were approved by the Institutional Review Board of the Institut National de la Santé et de la Recherche Médicale (IRB Inserm no. 0000388FWA00005831), and the Commission Nationale de l’Informatique et des Libertés (CNIL no. 908 450 and no. 909 216). It is registered at ClinicalTrials.gov with the number NCT03335644. Electronic informed consent was signed by all participants at inclusion.

### Data collection

#### Assessment of mindful eating (ME)

ME was assessed from March to September 2023 using the French version of the Mind-Eat Scale (https://info.etude-nutrinet-sante.fr/upload/siteinfo/protected/Quest_Alimentation_sensations_V5.pdf), a validated 24-item self-report questionnaire^21^. This scale features one overall dimension of ME and six sub-dimensions: awareness (e.g., “When I eat, I take the time to savor my foods”), non-reactivity (e.g., “When I see foods that I love, I find it hard not to eat them” (reversed)), openness (e.g., “I like to choose unfamiliar foods (meals at home or when out”)), gratitude (e.g., “I am grateful to the people who prepared the food I eat”), non-judgment (e.g., “I blame myself if I’ve eaten more than my body needs” (reversed)), and hunger/satiety (e.g., “I trust my body to know when to stop eating”), each of which includes four items. Each item is rated on a Likert scale from 1 (never or almost never) to 5 (always or almost always). The score for the global ME scale and each sub-dimension is obtained by adding individual scores of the respective items (after reverse-coding of the appropriate items) and dividing by the number of items. The final scores range from 1 (low ME) to 5 (high ME). The Mind-Eat Scale showed good internal consistency for the global dimension and all sub-dimensions (Cronbach’s α ranging from 0.78 to 0.91).

### Assessment of food intake

At inclusion and every 6 months afterward, participants were asked to complete a set of three 24-hour dietary records (randomly distributed between 2 weekdays and 1 weekend day, not necessarily consecutive). In order to obtain an adequate representation of food consumption, participants were selected if they completed at least one set of three 24-hour dietary records during a period of two years before completing the Mind-Eat Scale and ten months after (end of available data). Participants were instructed to self-report on the NutriNet website each food and drink consumed for each meal and occasion during the selected day and to estimate the portion sizes consumed using validated photographs^22^. Participants were asked to choose among seven portion sizes: three main portion sizes, two intermediate, and two extreme sizes. The average daily food intake was calculated and weighted according to the type of day (weekday or weekend). Energy intake was estimated using the published NutriNet-Santé food composition database, which contains more than 3,500 items^23^. Under-reporters were excluded and identified using the method proposed by Black, using the basal metabolic rate (BMR) and Goldberg cut-off points^24^. The validity of the dietary records in the NutriNet-Santé study has been shown by comparing the dietary records with biomarkers^25,26^ and with interviews by a dietitian^27^.

### Contribution of plant-based foods to the diet

The contribution of plant-based foods to the diet was assessed with the Plant-based Diet Index (PDI) score^5^. To compute this score, each food and drink item was classified into one of 18 food groups: 7 healthy plant-based food groups (whole-grain products, fruit, vegetables, nuts, legumes, vegetable oils, and tea/coffee), 5 less healthy plant-based food groups (fruit juices, refined grains, potatoes, sugar and sweetened beverages, sweets and desserts), and 6 animal food groups (animal fat, dairy, egg, fish or seafood, meat (poultry and red meat), miscellaneous animal food groups (e.g., pizza, mayonnaise or other creamy salad dressings)^4^. The original classification^5^ was adapted for the NutriNet-Santé database, to better match French consumption. Using data collected from the 24-hour dietary records, quintiles of consumption were calculated for each of the 18 food groups. For the 12 plant-based food groups, one to five points were given to increasing quintiles of consumption. This pattern of scoring was reversed for the 6 animal food groups. Scores of the plant-based food groups and the animal food groups were then summed to obtain the PDI. The theoretical PDI score ranges from 18 (low contribution of plant-based foods) to 90 (high contribution of plant-based foods). We also investigated the association between ME and sub-scores of PDI i.e. Healthy Plant-based Diet Index (hPDI), and Unhealthy Plant-based Diet Index (uPDI). In calculating the hPDI, the pattern of scoring was reversed for less healthy plant-based food groups, as for animal food groups, whereas for the uPDI, the pattern of scoring was reversed for healthy plant-based food groups, as for animal food groups^4^.

### Proportion of meat, fish, and dairy products in the diet

The proportions of meat (red meat, white meat and poultry, processed meat, and offal), fish (fish, shellfish, processed fish, and seafood), and dairy products (cheese, cottage cheese, *Petit-suisse*, milk, and yoghurt) in the diet were calculated using the 24-hour dietary records, by summing the quantity of specific food group consumption (in grams) divided by the total quantity of food (in grams) consumed (excluding water).

### Participants’ diet groups

To classify participants according to their diets, we used a mixed method combining 2 types of information: self-reported diet declarations and detailed dietary intake data using the 24-hour records^18^. First, participants were classified into three groups according to their self-reported diet: meat/fish eaters, vegetarians (exclusion of all meat and fish), or vegans (exclusion of all animal-based foods) according to self-reported diet from a “Food Choice” questionnaire (completed in 2022) or an “Anthropometric and Diet” questionnaire (completed each year since inclusion). Second, self-reported diets were compared with dietary data to verify whether participants who self-reported any type of vegetarianism or veganism were classified in the correct diet. The mean daily consumption of animal-based food groups (meat (e.g., red meat, poultry, and offal), processed meat, fish and other seafood, eggs, milk, cheese, plain yogurts, and milky desserts) in each category was assessed using the 24-hour records. Individuals who identified with a specific diet but consumed more than half of a portion of an animal-based food group excluded by this diet were reclassified into the appropriate category. The cutoffs for these animal-based food groups were defined as half a portion (e.g., 50 g/d of meat), using portion sizes from the French Programme National Nutrition Santé Guideline Score^28^. This method was used to avoid the misclassification of participants who deviated slightly from their usual diet.

Finally, we refined the group of meat/fish eaters into three subgroups: higher meat eaters (PDI < median), lower meat eaters (PDI ≥ median), and pesco-vegetarians (if the average meat consumption was ≤ to a half-portion of meat i.e. 50 g). In the end, all participants were classified into one of the five following groups ranging from highest meat intake to total avoidance of animal products: higher meat eaters, lower meat eaters, pesco-vegetarians, vegetarians, and vegans.

### Covariates

Potential confounders of the relationship between ME and food consumption were collected. We used the most recent data available at the time the Mind-Eat Scale was completed. The following confounders were selected: sex (male, female), age (years), educational level (primary, secondary, undergraduate, postgraduate), occupational status (unemployed, student, self-employed including farmer, employee and manual worker, intermediate profession, managerial staff and intellectual profession, retired), monthly income per household unit, smoking status (current/former/never), physical activity level (low/moderate/high), number of filled 24-hour dietary records, and energy intake (including alcohol). Body Mass Index (BMI < 18.5 / 18.5 ≤ BMI < 25.0 / 25.0 ≤ BMI < 30.0 / BMI ≥ 30) (kg/m2), cognitive restraint, and anxiety were also considered in sensitivity analyses as potential confounders. The number of people in the household was converted into several consumption units (CU) according to the National Institute for Statistics and Economic Studies (INSEE): 1 CU is attributed to the first adult in the household, 0.5 to other persons aged 14 and older and 0.3 for children under 14^29^. Monthly income categories were defined as follows: <1,200 / 1,200-1,799 / 1,800-2,699 / >2,700 euros per household unit, and “unwilling to answer”. Physical activity was assessed using the short form of the French version of the International Physical Activity Questionnaire (IPAQ)^30^. Weekly energy expenditure, expressed in Metabolic Equivalent of Task (MET) in minutes per day, was estimated, and three levels of physical activity were defined: low, moderate, and high. Cognitive restraint was assessed using the TFEQ-R21, which measures cognitive restraint, emotional eating, and uncontrolled eating^31^, with a score ranging from 6 (low cognitive restraint) to 24 (high cognitive restraint). Anxiety was assessed using the STAI-T, which measures trait anxiety^32^, with a score ranging from 20 (low anxiety) to 80 (high anxiety).

### Statistical analysis

To compare the characteristics of included with excluded participants, we performed Student t-tests for continuous variables and Pearson’s chi-square tests for categorical variables. We assessed the reliability of the Mind-Eat Scale by calculating Cronbach’s alpha (α). To investigate the relationship between participants’ characteristics and ME total score, we used Pearson correlation coefficients (with 95% CI) for continuous variables and Student t-test or ANOVA for categorical variables, as appropriate.

Multivariable linear regressions were used to analyze the association between ME (total and sub-dimensions) (independent variable) and PDI, hPDI, uPDI, and the proportion of meat, fish, and dairy products in the diet (dependent variables). Polytomous logistic regressions were used to analyze the association between ME (independent variable) and participants’ diet groups (dependent variable) classified into five modalities: higher meat eaters (PDI lower than the median), as the reference group, lower meat eaters (PDI above or equal to the median), pesco-vegetarians, vegetarians, and vegans.

Analyses were conducted on the whole sample as interactions between sex and ME were not significant for the large majority of variables. A minimally adjusted model was performed, controlling for sex and age. The main model was adjusted for sex, age, education level, occupational status, monthly income per household unit, smoking status, physical activity level, number of filled 24-hour dietary records, and energy intake. Further, sensitivity analyses were performed with an additional adjustment for Body Mass Index (BMI), cognitive restraint, or anxiety in the model. All tests of statistical significance were 2-sided, and significance was set at 5%. Missing data on confounders were handled with multiple imputations by a fully conditional specification (20 imputed datasets). Statistical analyses were performed using SAS software (SAS Institute Inc., version 9.4).

## Results

### Sample characteristics

From the NutriNet-Santé sample, 28,857 participants completed the Mind-Eat Scale, of whom 14 participants were excluded due to straightlining (i.e., consistently selecting the same modality). Among those, we selected the 13,768 participants who also completed at least three valid 24-hour dietary records in order to obtain a better representation of food consumption^33^.

Compared with excluded participants (those who presented straightlining and/or did not have at least three valid 24-hour dietary records), the 13,768 included participants were older (62.46 ± 13.42 years for included participants vs. 57.93 ± 14.18 years for excluded participants, *P* < 0.0001), had a higher proportion of males (28.35% vs. 22.90%, *P* < 0.0001), and a lower proportion of individuals with a high education level (39.64% vs. 41.70%, *P* = 0.002).

Table 1 shows the individual characteristics of the sample and a comparison of the ME score between them. On average, ME was positively associated with age and was higher in males, in retired participants, in participants with a higher income, and in participants with a higher physical activity. No difference according to education level and smoking status was found.

**Table 1:** Individual characteristics of the 13,768 participants and comparison of the mindful eating (ME) score according to these characteristics (NutriNet-Santé study, 2023).

	% or Mean (SD)	Mindful eating score^1^	*P*-value^2^
Age (years)	62.46 (13.42)		**< 0.0001**
Sex (%)			**< 0.0001**
Female	71.65	3.34 (0.53)	
Male	28.35	3.40 (0.46)	
Education level (%)			0.27
Primary	1.78	3.39 (0.48)	
Secondary	26.76	3.35 (0.50)	
Undergraduate	31.22	3.36 (0.52)	
Postgraduate	39.64	3.36 (0.52)	
Missing data	0.60	3.49 (0.49)	
Occupational status (%)			**< 0.0001**
Unemployed	5.19	3.36 (0.57)	
Student	0.38	3.24 (0.47)	
Self-employed, farmer	1.18	3.38 (0.60)	
Employee, manual worker	9.38	3.20 (0.53)	
Intermediate professions	10.10	3.28 (0.52)	
Managerial staff, intellectual professions	18.11	3.31 (0.54)	
Retired	55.59	3.42 (0.49)	
Missing data	0.07	3.40 (0.38)	
Monthly income (%)			**0.006**
<1200€	5.28	3.31 (0.58)	
1200–1799€	15.42	3.35 (0.52)	
1800–2699€	28.45	3.35 (0.52)	
≥ 2700€	38.99	3.37 (0.50)	
Unwilling to answer	11.77	3.37 (0.51)	
Missing data	0.09	3.63 (0.53)	
Smoking status (%)			0.55
Current	4.92	3.37 (0.52)	
Former	52.06	3.36 (0.50)	
Never	43.01	3.36 (0.53)	
Physical activity (%)			**< 0.0001**
Low	15.22	3.26 (0.53)	
Moderate	40.05	3.33 (0.51)	
High	44.73	3.42 (0.51)	
Energy intake (kcal)	1720.68 (476.86)		**0.008**
Number of 24-h dietary records	4.94 (1.39)		**0.006**
BMI, kg/m^2^ (%)			**< 0.0001**
< 18.5	5.45	3.58 ± 0.54	
18.65–24.99	61.58	3.42 ± 0.50	
25–29.99	24.08	3.24 ± 0.49	
≥ 30	8.89	3.09 ± 0.53	
Missing data	0.13	3.63 ± 0.63	
Cognitive restraint^3^	12.98 (3.60)		**< 0.0001**
Anxiety^4^	35.88 (10.30)		**< 0.0001**

Multiple imputation method is used to address missing data in the linear and logistic regression analyses.

Significant values are in bold.

^1^Measured with the Mind-Eat Scale. Score ranges from 1 to 5. A higher score corresponds to a higher mindful eating level.

^2^P-values based on Pearson correlation coefficients for continuous variables and Student t-test or ANOVA for categorical variables, as appropriate.

^3^Measured with the TFEQ-R21 questionnaire. Score ranges from 6 to 24. A higher score corresponds to a higher level of cognitive restraint.

^4^Measured with the STAI-T questionnaire. Score ranges from 20 to 80. A higher score corresponds to a higher anxiety level.

Table 2 shows the descriptive characteristics of ME scores (total and sub-dimensions), PDI, hPDI, and uPDI, proportions of meat, fish, and dairy consumption, and the distribution across the different participants’ diet groups in the sample.

**Table 2:** Descriptive characteristics of mindful eating (ME) and food intake in the 13,768 participants of the (NutriNet-Santé study, 2023).

Variables	% or Mean ± SD
Independent variable	
ME (total score)^1^	3.36 ± 0.52
Awareness	3.79 ± 0.79
Non-reactivity	3.16 ± 0.78
Openness	3.39 ± 0.89
Gratitude	3.16 ± 0.99
Non-judgement	3.13 ± 0.83
Hunger/satiety	3.53 ± 0.90
Dependent variables	
Plant-based Diet Index (PDI)^2^	51.32 ± 6.87
Healthy Plant-based Diet Index (hPDI)	55.43 ± 7.65
Unhealthy Plant-based Diet Index (uPDI)	54.49 ± 6.89
Meat consumption (%)	5.85 ± 4.01
Fish consumption (%)	2.78 ± 2.80
Dairy consumption (%)	11.91 ± 8.54
Diets (%)	
Higher meat eaters (PDI < 51)	36.99
Lower meat eaters (PDI ≥ 51)	30.85
Pesco-vegetarians	26.37
Vegetarians	4.79
Vegans	1.00

^1^Measured with the Mind-Eat Scale. Score ranges from 1 to 5. A higher score corresponds to a higher mindful eating level.

^2^Plant-based Diet Index score (PDI), healthy Plant-based Diet Index (hPDI), and unhealthy Plant-based Diet Index (uPDI) range from 18 to 90, higher scores respectively correspond to a higher plant-based food consumption, healthy plant-based food consumption, and unhealthy plant-based food consumption.

Table 3 shows the association between the total ME score and PDI, hPDI, uPDI, meat, fish, and dairy consumption, and participants’ diet groups (main model). Individuals with higher levels of total ME had higher PDI and hPDI scores (*P* < 0.0001) but a lower uPDI score (*P* < 0.0001). They also consumed a lower proportion of meat (*P* < 0.0001) and dairy products (*P* < 0.0001), while no association was observed with fish consumption (*P* = 0.38). Concerning the participants’ diet groups, individuals with higher levels of ME were more likely to be lower meat eaters, pesco-vegetarians, vegetarians, or vegans compared to individuals with lower levels of ME (*P* < 0.0001). Results for the minimally adjusted model are shown in **Supplemental Table 1.**

**Table 3:** Association between mindful eating (Mind-Eat Scale) and food intake in 13,768 participants (NutriNet-Santé study, 2023).

	Mindful eating (total score)	
	Beta-coefficients (95% CI)	*P*-value^1^
Plant-based Diet Index (PDI)^2^	1.19 (0.98, 1.41)	**< 0.0001**
Healthy Plant-based Diet Index (hPDI)	1.00 (0.76, 1.24)	**< 0.0001**
Unhealthy Plant-based Diet Index (uPDI)	– 0.48 (– 0.70, – 0.27)	**< 0.0001**
Meat consumption (%)	– 0.63 (– 0.76, – 0.50)	**< 0.0001**
Fish consumption (%)	– 0.04 (– 0.13, 0.05)	0.38
Dairy consumption (%)	– 0.86 (– 1.14, – 0.58)	**< 0.0001**

	OR (95% CI)	*P*-value^3^
Diets, %		
Higher meat eaters (PDI < 51)	Ref.	
Lower meat eaters (PDI ≥ 51)	1.13 (1.04, 1.23)	**0.004**
Pesco-vegetarians	1.56 (1.33, 1.83)	**< 0.0001**
Vegetarians	2.19 (1.57, 3.05)	**< 0.0001**
Vegans	1.35 (1.24, 1.48)	**< 0.0001**

*CI* Confidence Intervals, *OR* Odds Ratio.

Main model: adjusted for sex, age, educational level, occupational status, monthly household income, smoking status, physical activity, number of 24-hour dietary questionnaires, and dietary energy intake.

Significant values are in bold.

^1^P-value based on multivariable linear regression with mindful eating as a continuous independent variable and food intake as continuous dependent variables.

^2^Plant-based Diet Index score (PDI), healthy Plant-based Diet Index (hPDI), and unhealthy Plant-based Diet Index (uPDI) range from 18 to 90, higher scores respectively correspond to a higher plant-based food consumption, healthy plant-based food consumption, and unhealthy plant-based food consumption.

^3^P-value based on polytomous logistic regression with mindful eating as a continuous independent variable and food intake as a categorical dependent variable.

Table 4 shows the association between sub-dimensions of ME and food intake. The results observed for the total score of ME were mostly consistent across all sub-dimensions of ME with a few differences as follows. Awareness showed no association with diet groups. Non-reactivity was not associated with uPDI or dairy consumption but was associated with fish. Openness was associated with fish but not with a pesco-vegetarian diet. Non-judgment was not associated with hPDI, dairy consumption or lower meat eaters. Finally, hunger/satiety was not associated with hPDI, higher meat eaters or vegetarians, but was associated with fish. Overall, the dimensions of openness and gratitude consistently showed stronger associations with all outcomes compared to other dimensions.

Sensitivity analyses **(Supplemental Table 2)** showed consistent findings with the principal analysis after adjusting for cognitive restraint, anxiety, or BMI, although the association with lower meat eaters became non-significant, and effect sizes were slightly attenuated with BMI adjustment.

**Table 4:** Association between mindful eating (ME) sub-dimensions (Mind-Eat Scale) and food intake in 13,768 participants (NutriNet-Santé study, 2023).

	Mindful eating (subdimensions)					
	Awareness Beta-coefficients (95% CI)	*P*-value^1^	Non-reactivity Beta-coefficients (95% CI)	*P*-value^1^	Openness Beta-coefficients (95% CI)	*P*-value^1^
Plant-based Diet Index (PDI)^2^	0.33 (0.19, 0.47)	**< 0.0001**	0.43 (0.29, 0.58)	**< 0.0001**	0.53 (0.40, 0.65)	**< 0.0001**
Healthy Plant-based Diet Index (hPDI)	0.38 (0.23, 0.54)	**< 0.0001**	0.27 (0.12, 0.43)	**0.0006**	0.65 (0.51, 0.79)	**< 0.0001**
Unhealthy Plant-based Diet Index (uPDI)	– 0.40 (– 0.54, – 0.26)	**< 0.0001**	0.10 (– 0.04, 0.25)	0.15	– 0.72 (– 0.84, – 0.59)	**< 0.0001**
Meat consumption (%)	– 0.10 (– 0.19, – 0.02)	**0.020**	– 0.37 (– 0.47, – 0.29)	**< 0.0001**	– 0.14 (– 0.21, – 0.06)	**0.0003**
Fish consumption (%)	0.05 (– 0.01, 0.11)	0.08	– 0.13 (– 0.19, – 0.07)	**< 0.0001**	0.12 (0.07, 0.17)	**< 0.0001**
Dairy consumption (%)	– 0.46 (– 0.64, – 0.27)	**< 0.0001**	0.12 (– 0.07, 0.30)	0.21	– 0.36 (– 0.52, – 0.20)	**< 0.0001**

	OR (95% CI)	*P*-value ^3^	OR (95% CI)	*P*-value ^3^	OR (95% CI)	*P*-value ^3^
Diets (%)						
Higher meat eaters (PDI < 51)	Ref.		Ref.		Ref.	
Lower meat eaters (PDI ≥ 51)	1.00 (0.95, 1.06)	0.90	1.05 (0.99, 1.10)	0.11	1.07 (1.02, 1.13)	**0.0038**
Pesco-vegetarians	1.10 (0.99, 1.23)	0.07	1.37 (1.23, 1.53)	**< 0.0001**	0.94 (0.86, 1.03)	0.19
Vegetarians	1.13 (0.91, 1.41)	0.27	1.59 (1.27, 1.98)	**< 0.0001**	1.42 (1.16, 1.73)	**0.0006**
Vegans	1.05 (0.99, 1.11)	0.12	1.18 (1.12, 1.25)	**< 0.0001**	1.07 (1.02, 1.12)	**0.0089**

	Mindful eating (subdimensions)					
	Gratitude Beta-coefficients (95% CI)	*P*-value^1^	Non-judgment Beta-coefficients (95% CI)	*P*-value^1^	Hunger/Satiety Beta-coefficients (95% CI)	*P*-value^1^
Plant-based Diet Index (PDI)^2^	0.54 (0.43, 0.65)	**< 0.0001**	0.30 (0.17, 0.44)	**< 0.0001**	0.32 (0.20, 0.45)	**< 0.0001**
Healthy Plant-based Diet Index (hPDI)	0.59 (0.46, 0.71)	**< 0.0001**	0.04 (– 0.11, 0.18)	0.63	0.07 (– 0.06, 0.21)	0.29
Unhealthy Plant-based Diet Index (uPDI)	– 0.27 (– 0.38, – 0.15)	**< 0.0001**	0.13 (0.00, 0.27)	**0.049**	0.19 (0.07, 0.32)	**0.002**
Meat consumption (%)	– 0.22 (– 0.29, – 0.16)	**< 0.0001**	– 0.24 (– 0.33, – 0.17)	**< 0.0001**	– 0.25 (– 0.32, – 0.17)	**< 0.0001**
Fish consumption (%)	– 0.04 (– 0.09, 0.00)	0.06	– 0.04 (– 0.09, 0.02)	0.22	– 0.05 (– 0.11, – 0.00)	**0.04**
Dairy consumption (%)	– 0.46 (– 0.60, – 0.32)	**< 0.0001**	– 0.11 (– 0.28, 0.06)	0.22	– 0.42 (– 0.58, – 0.26)	**< 0.0001**

	OR (95% CI)	*P*-value^3^	OR (95% CI)	*P*-value^3^	OR (95% CI)	*P*-value^3^
Diet groups (%)						
Higher meat eaters (PDI < 51)	Ref.		Ref.		Ref.	
Lower meat eaters (PDI ≥ 51)	1.09 (1.04, 1.13)	**0.0002**	1.03 (0.98, 1.08)	0.31	1.01 (0.96, 1.06)	0.73
Pesco-vegetarians	1.29 (1.19, 1.40)	**< 0.0001**	1.13 (1.02, 1.24)	**0.016**	1.26 (1.14, 1.38)	**< 0.0001**
Vegetarians	1.41 (1.18, 1.67)	**0.0001**	1.35 (1.10, 1.65)	**0.0034**	1.12 (0.93, 1.36)	0.24
Vegans	1.13 (1.09, 1.19)	**< 0.0001**	1.12 (1.06, 1.18)	**< 0.0001**	1.13 (1.07, 1.18)	**< 0.0001**

*CI* Confidence Intervals, *OR* Odds Ratio.

Main model: adjusted for sex, age, educational level, occupational status, monthly household income, smoking status, physical activity, number of 24-hour dietary questionnaires, and dietary energy intake.

^1^P-value based on multivariable linear regression with mindful eating as a continuous independent variable and food intake as a dependent variable.

^2^Plant-based Diet Index score (PDI), healthy Plant-based Diet Index (hPDI), and unhealthy Plant-based Diet Index (uPDI) range from 18 to 90, higher scores respectively correspond to a higher plant-based food consumption, healthy plant-based food consumption, and unhealthy plant-based food consumption.

^3^P-value based on polytomous logistic regression with mindful eating as a continuous independent variable and food intake as a dependent variable.

## Discussion

In this study, we found that individuals with higher levels of ME had a diet richer in plant-based foods, especially healthy ones, while their consumption of unhealthy plant-based foods was lower. In addition, individuals with higher levels of ME consumed less meat and dairy, but not less fish. These individuals were also more likely to be lower meat eaters, pesco-vegetarians, vegetarians, or vegans. Most of these associations were consistent across all sub-dimensions of ME, with few exceptions. Overall, openness and gratitude were the dimensions that showed the strongest associations.

To the best of our knowledge, no previous study has examined the relationship between an overall ME score and diets rich in plant-based foods. Only one study focused on the broader concept of mindfulness and showed higher affective attitudes towards vegetarian foods in individuals with higher levels of mindfulness^34^. Additionally, greater sustainable consumption, which can be achieved by reducing meat consumption and preferring plant-based foods^35,36^, has been repeatedly shown in individuals with higher levels of mindfulness^37^.

Investigating the sub-dimensions of ME is of particular interest, as it highlights the aspects of ME that are most crucial for plant-based food consumption and clarifies the mechanisms linking ME to dietary behavior, considering environmental, ethical, and health aspects. In our study, the total ME score showed consistent results across all sub-dimensions, with the strongest associations observed for gratitude and openness. This suggests that these dimensions of ME may have the greatest impact on plant-based food choices, particularly because of their link to environmental and ethical considerations. In addition, some discrepancies in the significance of associations with fish consumption, pesco-vegetarianism, vegetarianism, and veganism across subdimensions were noted. These inconsistencies may reflect a conflict between health and environmental concerns in the adoption of a more plant-based diet^38^.

The Gratitude dimension is defined as the positive appreciation for elements that have contributed to meals (e.g., people, planet)^21^. This subdimension showed associations with all dietary outcomes in our study, except for fish consumption. Individuals scoring higher in the health of the planet sub-dimension of the Expanded Mindful Eating Scale (EMES) which closely aligns with the gratitude sub-dimension of the Mind-Eat Scale, were more likely to adhere to a healthy plant-based dietary pattern, and less likely to adopt an unhealthy plant-based dietary pattern^14^, consistent with our findings. Gratitude has also been shown to be positively correlated with conscientiousness, openness, and emotional stability^39^, traits associated with a higher intake of plant-based foods^40^. Gratitude may lead to a more responsible purchasing of plant-based foods due to a greater understanding and appreciation of the origins of their food. Additionally, individuals with higher gratitude may also feel a greater responsibility towards future generations^41^, which has been associated with environmental concerns, such as increased pro-environmental intentions and concern about climate change^41^. Grateful individuals may also adopt a diet low in meat for ethical reasons, such as protecting animal welfare, which is a common motivation for choosing a plant-based diet^42,43^. This aligns with their tendency to exhibit higher levels of empathy^39,44^, a trait that is also observed to be higher in vegetarians^42,43^. Finally, the lack of association between ME and fish consumption may be attributed to the fact that humans exhibit greater empathy for mammals than for fish, which are less closely related by evolutionary history^45^.

The Openness dimension, which assesses open-mindedness and curiosity about food, was associated with all outcomes except pesco-vegetarianism. Consistently, a positive association was previously found between openness measured by the Five Factor Model of Personality (BIG 5) and the consumption of plant-based foods^40^. Individuals with an open mind, linked to intellectual curiosity^46^, may be more aware of the environmental necessity of reducing meat consumption in favor of plant-based alternatives and have a greater concern for animal welfare. Openness may also encourage individuals to consume less conventional foods and experiment with new recipes, which may include ingredients that are unfamiliar or less readily available in stores.

The Non-reactivity dimension was associated with all variables in our study, except for the unhealthy plant-based diet and lower meat eaters. Non-reactivity has been positively associated with sustainable behaviors in the literature. However, no study has yet explored the potential association between non-reactivity and the consumption of plant-based foods. By reducing emotional eating^47^, non-reactivity may promote healthier dietary patterns, including a higher intake of plant-based foods like fruits and vegetables^48^, and could even encourage a shift toward vegetarianism and veganism^49^. Individuals with higher levels of non-reactivity may also exhibit lower impulsivity^50^, which has been associated with a lower adherence to a healthy plant-based diet^51^. Such individuals may take more time to deliberate over their food choices, shop for and prepare specific food, and may be less inclined to adhere to traditional French meals that are often high in meat. They may prefer to avoid fast and easily accessible foods, instead taking the time to seek out vegetarian or vegan options available in specific recipe books and aisles of stores.

In our study, the Awareness dimension was associated with PDI, hPDI, uPDI, meat, and dairy consumption. In the literature, awareness assessed with the EMES questionnaire has also been positively associated with a higher plant-based diet and negatively associated with an unhealthy plant-based diet^14^. The mindfulness sub-dimension of acting with awareness has also been associated with sustainable behaviors^52^ and greenness^17^. Taking time to consider environmental, ethical, and health issues may represent a significant aspect of awareness and lead to healthier plant-based food consumption. Specifically, we hypothesize that these concerns are stronger in the case of meat, which may explain its association with ME, while no such association was observed for fish. Acting with awareness has been associated with greater responsibility^46^, possibly prompting these individuals to demonstrate greater concern for their health and the environment. Finally, an increased awareness may be associated with a greater connectedness to nature^16^, which in turn can lead to environmentally and ethically conscious choices.

In our study, the Hunger/Satiety dimension was associated with most outcomes, except for a healthy plant-based diet, lower meat eaters, and vegetarianism. There was no data in the literature on the association between this sub-dimension and a plant-based diet. However, poorer hunger-satiety-specific cues, assessed by the Intuitive Eating Scale-2, similar to this concept, have been linked to greater emotional eating^53^. Emotional eating, in turn, has been negatively associated with vegetarianism and veganism^49^, possibly explaining the relationship observed between hunger/satiety and overall consumption of plant-based food in our study. Interestingly, in our study, relying more on hunger/satiety cues was associated with greater consumption of plant-based foods, particularly unhealthy options, while showing no association with healthy choices. This suggests that these individuals may prioritize ethical concerns (altruistic) over health considerations (self-focused), even though unhealthy plant-based foods are not always more environmentally sustainable. However, despite increased consumption of unhealthy plant-based foods, individuals with higher scores on the hunger/satiety dimension maintain better overall diet quality, as indicated by previous results in the same cohort^54^.

The Non-judgment dimension was associated with most outcomes, except for hPDI, fish and dairy consumption, and lower meat eaters. Individuals with higher levels of non-judgment consumed more plant-based foods, particularly unhealthy options. Consistently, another study found a positive correlation between non-judgment, assessed by the EMES scale, and an unhealthy plant-based diet^14^. This could indicate a greater interest in environmental aspects than in health among participants with higher levels of non-judgment. Individuals with higher levels of non-judgment may exhibit higher levels of self-compassion, which can extend to environmental protection and ethical considerations. However, this self-compassion could occasionally lead to greater acceptance regarding the consumption of less healthy foods.

Our findings suggest a potential positive effect of ME in promoting the consumption of plant-based foods, most of the time healthy plant-based foods, which can benefit both human health and the environment. Integrating ME programs or components into interventions could represent a novel approach to promoting sustainable eating behaviors. Several ME interventional programs are already available^55,56^, focusing on aspects such as the awareness of food, physical hunger and satiety cues, environmental or emotional triggers to eat, and the acceptance of one’s body. However, these programs primarily focus on health primary evaluation criteria and give less attention to environmental and ethical considerations. Furthermore, it could be beneficial to consider incorporating the ME concept into French dietary guidelines, as seen in other countries, which have included messages like “Be mindful of your eating habits”^12^ or “Take your time eating and take a break. Slow and conscious eating also promotes the feeling of satiety”^13^. Including environmental and ethical considerations in these guidelines could further enhance their impact on promoting sustainable dietary behaviors.

To our knowledge, this is the first study to investigate and show an association between ME and a plant-based quality diet in a large sample. However, its cross-sectional design limits our ability to establish causality between ME and food intake, and reverse causality remains a possibility, as individuals adhering to more plant-based diets might subsequently develop higher levels of ME. It would be beneficial for future research to assess the impact of ME on plant-based diets in longitudinal or intervention studies. Another strength of the study is the large population-based sample with individuals of different socio-demographic characteristics and nutritional status, which provided great statistical power and allowed us to control for multiple confounders in our analyses. While the voluntary recruitment of participants suggests a strong interest in health and nutrition issues, this factor also ensures a highly engaged and motivated sample, enhancing the reliability of the data collected. Nevertheless, selection bias cannot be ruled out^27,57^, so caution should be exercised in generalizing our results to the broader French population. For example, our sample included more females, older individuals, and graduates with healthier diets compared to the general population^27^. The methods used to assess ME and dietary data are another major strength of the study. The validated Mind-Eat Scale has shown good psychometric properties and is the only scale available that allows the calculation of a total ME score^21^. Additionally, 24-hour dietary records are widely used in epidemiological research^58^ and provide a good representation of the participants’ food consumption. The use of at least two recordings has been shown to be one of the most appropriate ways of obtaining internationally comparable data^33^. In this study, we utilized three recordings to enhance accuracy. However, this method can lead to estimation errors and under-reporting of quantities consumed. For instance, participants aware of the recording date in advance may have adjusted their consumption unintentionally or intentionally^59^. Finally, PDI is a well-established diet quality index with a good construct validity^60^. However, it may at times arbitrarily assign foods. In addition, the scores distinguishing healthy from unhealthy plan-based diets may vary across studies^60^.

## Conclusion

Our study found that participants with higher levels of ME consumed more plant-based foods, particularly healthy ones, and were more likely to be vegetarian or vegan. The results for the ME sub-dimensions indicate that most facets of ME are associated with a healthy plant-based diet, particularly gratitude and openness. Overall, this study suggests that ME could be an effective tool for promoting healthy and environmentally friendly dietary changes, although the cross-sectional design limits inferences. Further, population-based studies, particularly longitudinal and intervention studies, are needed to confirm these findings and establish causality.

## Electronic supplementary material

Below is the link to the electronic supplementary material.

Supplementary Material 1

## Data Availability

The datasets used and/or analyzed during the current study are available from the corresponding author on reasonable request.

## Abbreviations

BMI: Body Mass Index
BMR: Basal Metabolic Rate
CI: Confidence Interval
CU: Consumption Unit
hPDI: Healthy Plant Diet Index
IPAQ: International Physical Activity Questionnaire
ME: Mindful Eating
MET: Metabolic Equivalent Task
PAL: Physical Activity Level
PDI: Plant Diet Index
uPDI: Unhealthy Plant Diet Index
UPFs: Ultra-processed Foods
