# Supplementary material for: Mindful eating is associated with a healthier plant-based diet in the NutriNet-Santé study
Source: Sci Rep. 2025 Jun 6;15:19928. doi: 10.1038/s41598-025-02195-5 (PMC12144130; doi:10.1038/s41598-025-02195-5)
Supplement: Supplementary file 1 — Supplementary Material 1 [file 41598_2025_2195_MOESM1_ESM.docx]

**Supplemental table 1.** Association between mindful eating (Mind-Eat Scale) and food intake in 13,768 participants (minimally adjusted model) (NutriNet-Santé study, 2023)

|  | **Mindful eating (total score)** | |
| --- | --- | --- |
|  | **Beta-coefficients (95% CI)** | **P-value^1^** |
| **Plant-based Diet Index (PDI)^2^** | 1.22 (1.00, 1.44) | **<0.0001** |
| Healthy Plant-Based Diet Index (hPDI) | 1.33 (1.09, 1.58) | **<0.0001** |
| Unhealthy Plant-Based Diet Index (uPDI) | -0.51 (-0.73, -0.29) | **<0.0001** |
| **Meat consumption, %** | -0.72 (-0.85, -0.59) | **<0.0001** |
| **Fish consumption, %** | -0.02 (-0.11, 0.07) | 0.69 |
| **Dairy consumption, %** | -0.85 (-1.13, -0.57) | **<0.0001** |
|  | **OR (95% CI)** | **P-value^3^** |
| **Diet groups, %** |  |  |
| Higher meat eaters (PDI < 51) | Ref |  |
| Lower meat eaters (PDI ≥ 51) | 1.12 (1.04, 1.22) | **0.0051** |
| Pesco-vegetarians | 1.68 (1.44, 1.98) | **<0.0001** |
| Vegetarians | 2.28 (1.64, 3.19) | **<0.0001** |
| Vegans | 1.43 (1.32, 1.56) | **<0.0001** |

Abbreviations: CI, Confidence Intervals; OR, Odds Ratio

Minimally-adjusted model: adjusted for sex and age.

^1^ P-value based on multivariable linear regression with mindful eating as a continuous independent variable and food intake as dependent variables.

^2^ Plant Diet Index score (PDI), healthy Plant Diet Index (hPDI), and unhealthy Plant Diet Index (uPDI) range from 18 to 90, higher scores respectively correspond to a higher plant-based food consumption, healthy plant-based food consumption, and unhealthy plant-based food consumption.

^3^ P-value based on polytomous logistic regression with mindful eating as a continuous independent variable and food intake as dependent variable.

**Supplemental table 2:** Association between mindful eating (MIND-EAT Scale) and food intake in 13,768 participants (sensitivity analysis) (NutriNet-Santé study, 2023)

|  | **Mindful eating (total score)** | | | | | |
| --- | --- | --- | --- | --- | --- | --- |
|  | **BMI** | | **Cognitive restraint** | | **Anxiety** | |
|  | **Beta-coefficients^1^ (95% CI)** | **P-value ^5^** | **Beta-coefficients^2^ (95% CI)** | **P-value ^5^** | **Beta-coefficients^3^ (95% CI)** | **P-value ^5^** |
| **Plant Diet Index (PDI)^4^** | 0.62 (0.41, 0.84) | **<0.0001** | 1.08 (0.86, 1.31) | **<0.0001** | 1.27 (1.05, 1.5) | **<0.0001** |
| Healthy Plant Diet Index (hPDI) | 0.43 (0.19, 0.68) | **0.0004** | 1.15 (0.90, 1.40) | **<0.0001** | 1.12 (0.86, 1.37) | **<0.0001** |
| Unhealthy Plant Diet Index (uPDI) | -0.48 (-0.70, -0.25) | **<0.0001** | -0.80 (-1.02, -0.57) | **<0.0001** | -0.47 (-0.70, -0.24) | **0.0001** |
| **Meat consumption, %** | -0.27 (-0.4, -0.14) | **0.0001** | -0.61 (-0.74, -0.47) | **<0.0001** | -0.68 (-0.81, -0.54) | **<0.0001** |
| **Fish consumption, %** | 0.04 (-0.05, 0.14) | 0.36 | 0.00 (-0.09, 0.10) | 0.98 | -0.06 (-0.15, 0.04) | 0.25 |
| **Dairy consumption, %** | -0.66 (-0.95, -0.37) | **<0.0001** | -0.72 (-1.05, -0.39) | **<0.0001** | -0.94 (-1.24, -0.64) | **<0.0001** |
|  | **OR (95% CI)** | **P-value ^6^** | **OR (95% CI)** | **P-value ^6^** | **OR (95% CI)** | **P-value ^6^** |
| **Diet groups, %** |  |  |  |  |  |  |
| Higher meat eaters (PDI < 51) | Ref |  | Ref |  | Ref |  |
| Lower meat eaters (PDI ≥ 51) | 1.02 (0.93, 1.11) | 0.73 | 1.11 (1.01, 1.22) | **0.036** | 1.10 (1.01, 1.21) | **0.04** |
| Pesco-vegetarians | 1.22 (1.03, 1.44) | **0.019** | 1.50 (1.23, 1.84) | **0.0001** | 1.73 (1.45, 2.07) | **<0.0001** |
| Vegetarians | 1.64 (1.17, 2.30) | **0.0044** | 1.62 (1.01, 2.59) | **0.043** | 2.65 (1.8, 3.91) | **<0.0001** |
| Vegans | 1.10 (1.00, 1.20) | **0.045** | 1.32 (1.19, 1.46) | **<0.0001** | 1.41 (1.28, 1.54) | **<0.0001** |

Abbreviations: CI, Confidence Intervals; OR, Odds Ratio

Models: adjusted for sex, age, educational level, occupational status, monthly household income, smoking status, physical activity, number of 24-hour dietary questionnaires, dietary energy intake, and BMI^1^, cognitive retraint^2^ (TFEQ), and anxiety^3^ (STAIT-T).

^4^ Plant Diet Index score (PDI), healthy Plant Diet Index (hPDI), and unhealthy Plant Diet Index (uPDI) range from 18 to 90, higher scores respectively correspond to a higher plant-based food consumption, healthy plant-based food consumption, and unhealthy plant-based food consumption.

^5^ P-value based on multivariable linear regression with mindful eating as a continuous independent variable and food intake as continuous dependent variables.

^6^ P-value based on polytomous logistic regression with mindful eating as a continuous independent variable and food intake as an ordinal dependent variable.
